# Characteristics of drug overdose suicide attempts presenting to the psychiatric emergency department of Beijing Anding Hospital

**DOI:** 10.1186/s12889-024-19095-4

**Published:** 2024-06-14

**Authors:** Lihui Tu, Yi Liu, Hui Zhu, Qinge Zhang, Xiao Ji

**Affiliations:** Beijing Key Laboratory of Mental Disorders, Beijing Anding Hospital, & the Advanced Innovation Center for Human Brain Protection, National Clinical Research Center for Mental Disorders & National Center for Mental Disorders, Capital Medical University, 5 Ankang Lane, Dewai Avenue, Xicheng District, Beijing, 100088 China

**Keywords:** Psychiatry emergency department, Attempted suicide, Overdose

## Abstract

**Background:**

Overdose-related suicide attempts represent a significant portion of self-harm presentations in the psychiatric emergency department (ED). Identifying specific patient characteristics associated with these attempts holds promise for pinpointing drug classes with elevated risk and paving the way for tailored suicide prevention interventions. This study aims to examine the demographic profiles of ED patients who had experienced overdose-related suicide attempts.

**Methods:**

This retrospective study was conducted at Beijing Anding Hospital, Capital Medical University, from January 2020 to December 2021. Patients with psychiatric drug overdose suicide attempts presenting to the psychiatric ED were included. Sociodemographic characteristics and the specific classes of drugs involved were collected, and analysed descriptively.

**Results:**

This study examined 252 overdose patients, excluding 51 patients treated with alcohol or nonpsychiatric drugs, and a total 201 cases were included. The mean age of the patients was 28 ± 16 years (median 23, range 12–78), and 82% (*n* = 165) of the sample were females. Notably, nearly half (45%) of the patients were aged ≤ 20 years. While the number of cases decreased with increasing age, a significant increase was observed in 2021 compared to 2020. Benzodiazepines (BZDs) were the most frequently implicated substance class (*n* = 126, 63%), followed by antidepressants (*n* = 96, 48%), antipsychotics (*n* = 44, 22%), Z-drugs (*n* = 43, 21%), and mood stabilizers (*n* = 36, 18%). For adolescents, antidepressants (*n* = 52, 71%) overtook BZDs (*n* = 38, 52%) as the most common drug. The monthly distribution of cases revealed peaks in April and November. Furthermore, 21% (*n* = 42) of patients ingested more than two psychotropic medications concurrently. Finally, approximately half (*n* = 92) of the patients required inpatient admission for further treatment. Comparisons between hospitalized and nonhospitalized patients did not reveal any significant differences.

**Conclusions:**

The present study revealed a greater prevalence of suicide overdose attempts among young females receiving prescriptions for antidepressants and/or BZDs. This finding suggests a potential need for enhanced monitoring of suicidal behaviour in this specific population when prescribing psychotropic medications. These findings contribute to the growing body of knowledge regarding drug overdose suicide attempts in psychiatric emergency settings and underscore the importance of further research to develop targeted prevention interventions.

**Supplementary Information:**

The online version contains supplementary material available at 10.1186/s12889-024-19095-4.

## Introduction

Overdose suicide represents a significant public health concern, particularly in individuals with mental disorders [1–3]. Data [4] from a single Northern Chinese general hospital’s emergency department (ED) admissions for acute poisoning suggest that over half of the drug poisoning cases were attributable to suicide attempts. Sedative-hypnotics were the most common primary agent used, aligning with findings from Western countries (Switzerland, Ireland, and Northern Ireland) [5, 6]. However, research within psychiatric settings exploring this phenomenon has been limited. In psychiatric patients, both our prior research and a recent independent investigation have implicated anxiolytics as a common drug class associated with suicide attempts by overdose [7–9]. These findings suggest a potential similarity in presenting patterns regarding overdose suicide attempts across general and psychiatric healthcare settings.

The convergence of evolving psychiatric drug therapies and the ongoing COVID-19 pandemic may contribute to a more complex landscape of overdose suicide. In-depth characterization of psychiatric patients engaging in drug overdose suicide attempts is crucial for identifying high-risk individuals and enabling early intervention, a cornerstone of suicide prevention. This study therefore analysed the characteristics of patients who had undergone psychiatric drug overdose suicide attempts in the psychiatric ED over the past two years (2020–2021). The primary objective of this research was to characterize potential high-risk patients for suicide attempts within the psychiatric population. This knowledge could guide the development of targeted and effective suicide prevention measures.

## Materials and methods

### Study design

This retrospective study was conducted at the ED of Beijing Anding Hospital, spanning from January 1, 2020, to December 31, 2021. Beijing Anding Hospital, a psychiatric hospital specializing in mental health care, established its ED in 2008. However, the hospital has provided emergency psychiatric care since 1952. Prior to the COVID-19 pandemic, it served as the sole psychiatric ED in northern China. Currently, it remains a primary center for managing psychiatric emergencies within the capital and surrounding regions of northern, northeastern, and northwestern China. As of 2021, the ED received nearly 22,000 admissions, averaging approximately 1,900 cases per month. The study, approved by the hospital ethics committee (Approval No. 2022-202314FS-2), waived the need for informed consent given its retrospective design and adherence to routine care protocols. It encompassed patients of all ages presenting to the emergency department after drug-induced suicide attempts.

This study included patients who presented to the psychiatric ED following a suicide attempt by drug overdose and subsequently underwent a drug toxicity test after receiving emergency treatment. Individuals with multiple recorded suicide attempts during the study period were counted as a single case for the present analysis. Consistent with the findings of previous research [10, 11], an attempted suicide was defined as any self-injurious behaviour with the intent to end one’s life that did not result in death. The exclusion criteria for individuals in the sample were unintentional overdoses (e.g., medication misuse due to illness or recreational drug use without suicidal intent), nondrug poisons (e.g., chemicals), or overdoses solely involving alcohol, regardless of intent.

The following data were collected for each patient: demographic data (gender, date of birth), medication time and date (hour of overdose categorized as daytime [8:00 AM − 7:00 PM], early-night [7:00 PM − 00:00 AM], or late-night [00:00 AM − 8:00 AM]), weekends/weekday status (including legal holidays), drug toxicity test results, specific types and number of drugs involved (categorized as psychotropic drugs [antipsychotics, antidepressants, benzodiazepines (BZDs), Z-drugs, mood stabilizers] or nonpsychotropic drugs), concomitant alcohol use, and aftercare (hospitalization or not). Additionally, data on COVID-19 pandemic-related prevention and control measures were recorded for analysis.

### Statistical analysis

To exam the characteristics of different age groups, patients were categorized into ten-year intervals (≤ 20, 21–30, 31–40, 41–50, 51–60, > 60 years old). The age distribution was then evaluated by sex and year separately. Additionally, the distribution of common psychotropic medications used was examined by sex and across the different age groups. Finally, the monthly distribution of ED presentations was investigated to assess potential seasonal patterns, particularly among adolescents.

Subgroup analyses were conducted to gain a comprehensive understanding of the data. First, comparisons between 2020 and 2021 were performed to identify potential changes over time. Second, adolescents aged 12–18 years comprised over one-third of the sample. Therefore, this subgroup was analyzed separately. Additionally, patients with different aftercare outcomes were compared to assess whether those requiring hospitalization presented with greater severity of illness.

Descriptive statistical analyses were performed using Microsoft Excel and SPSSsoftware (version 26.0; IBM SPSS Statistics, Chicago, IL, USA). Age, the only continuous variable, was not normally distributed according to the Kolmogorov-Smirnov test. Therefore, it is presented as the medians and interquartile ranges (IQRs), and analysed using the Mann–Whitney-U Test. Categorical variables are presented as frequencies and percentages (%). Chi-square tests were used to analyse these variables. A two-sided *P* value < 0.05 was considered statistically significant.

## Results

### Demographic of attempts

**Fig. 1:**
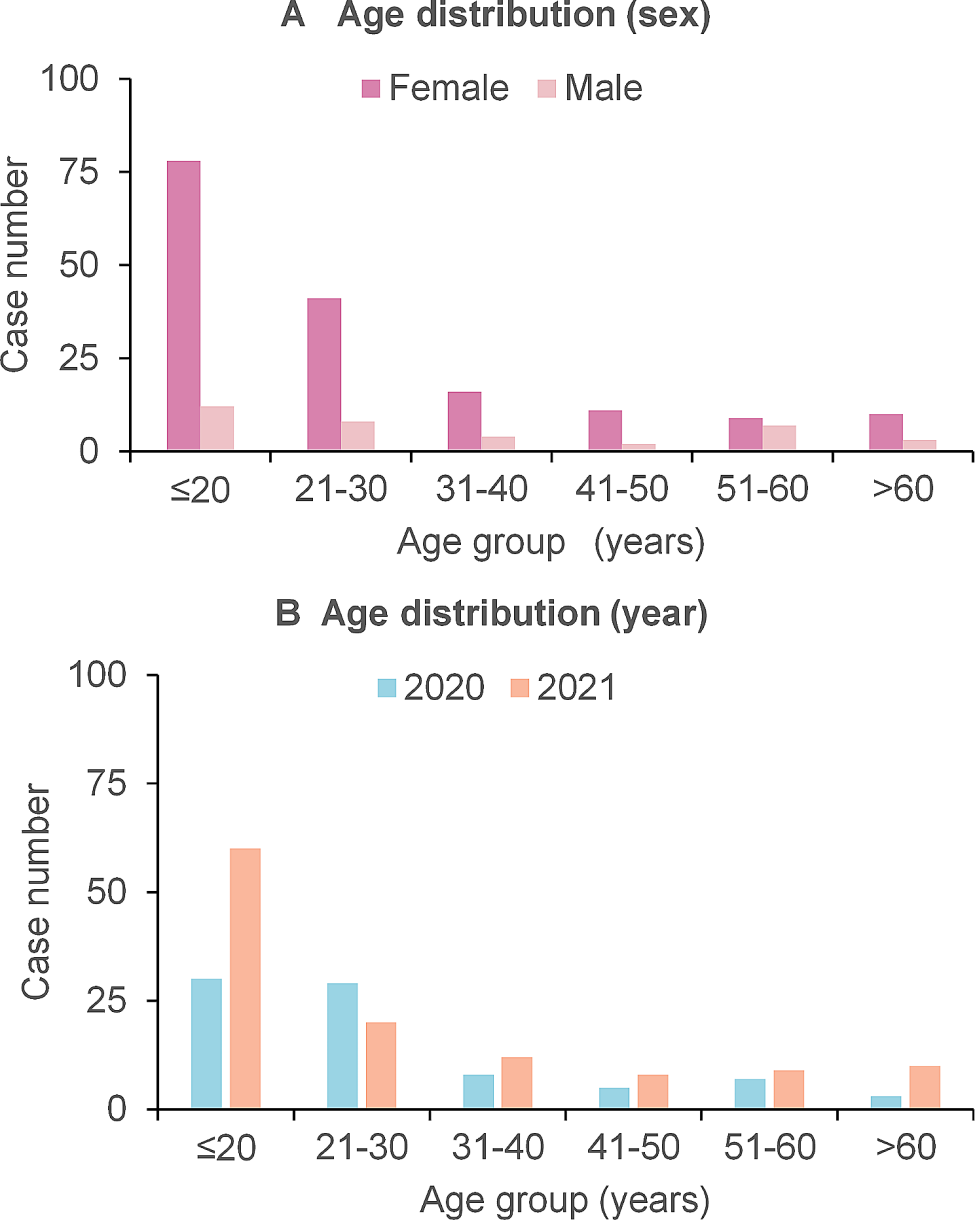
Age distribution by (**a**) sex and (**b**) year

Between January 2020 and December 2021, a total of 252 patients presented to the psychiatric ED after overdosing on drugs and subsequently underwent drug toxicity testing. After applying the inclusion criteria, 201 patients (79.8%) were ultimately included in the study. The mean age of the study population was 28 ± 16 years (median 23, range 12–78), with females comprising 82% (*n* = 165) of the sample. Notably, nearly half (45%) of the patients were aged ≤ 20 years. While the number of cases generally decreased with increasing age, a significant increase was observed in 2021 compared to 2020. This upwards tendency in overdose presentations coincided with the increasing number of COVID-19 patients documented during the same period (Fig. 1, Supplementary Figure/Table 1). Comparisons between 2020 and 2021 yielded largely consistent results in terms of clinical characteristics and most drug classes involved. However, a significantly higher proportion of males was observed in 2021 (*P* = 0.033). Additionally, a trend towards younger individuals in 2021 was noted, although this difference did not reach statistical significance (50% vs. 37%, *P* = 0.057). Further details regarding the characteristics of the included patients are presented in Table 1.

**Table 1:** The main characteristics of patients who presented due to attempted suicide with drugs

Variables		Number of Cases (%)		
	Total (*N* = 201)	Year 2020 (*n* = 82)	Year 2021 (*n* = 119)	*P*
Female	165 (82)	73(89)	92(77)	**0.033**
Age group (years)				
Median (IQRs)	23(19)	24(17)	20(21)	0.496
≤ 20	90(45)	30(37)	60(50)	
21–30	49(24)	29(35)	20(17)	
31–40	20(10)	8(10)	12(10)	
41–50	13(7)	5(6)	8(7)	0.057
51–60	16(8)	7(9)	9(8)	
> 60	13(7)	3(4)	10(8)	
Medication time^*^				0.342
Daytime	74(37)	31(38)	43(36)	
Early-night	36(18)	11(13)	25(21)	
Late-night	34(17)	16(20)	18(15)	
Medication on weekends/holidays	46(23)	18(22)	28(24)	0.794
Concomitant use of alcohol	10(5)	6(7)	4(3)	0.348
≥ 3 drugs consumption	42(21)	17(21)	25(21)	0.962
Aftercare: hospitalization	92(46)	33(40)	59(50)	0.192
Psychotropic drugs				
BZDs	126(63)	56(68)	70(59)	0.173
Antidepressants	96(48)	38(46)	58(49)	0.828
Antipsychotics	44(22)	15(18)	29(24)	0.306
Z-drugs	43(21)	25(31)	18(15)	**0.009**
Mood stabilizers	36(18)	19(23)	17(14)	0.215
Nonpsychotropic drugs	39(19)	14(17)	25(21)	0.488

*57 patients are missing; IQRs, interquartile ranges BZDs, benzodiazepines

### Characteristics of overdose drugs

The five most commonly overdosed drug classes were BZDs (*n* = 126, 63%), antidepressants (*n* = 96, 48%), antipsychotics (*n* = 44, 22%), Z-drugs (*n* = 43, 21%), and mood stabilizers (*n* = 36, 18%). Nonpsychotropic drugs, including trihexyphenidyl, tandospirone, traditional Chinese medicine (TCM), and medications for body ailments, accounted for 19% of the patients. Alcohol use was infrequent, though a few patients received potentially fatal combinations of antibiotics.

**Fig. 2:**
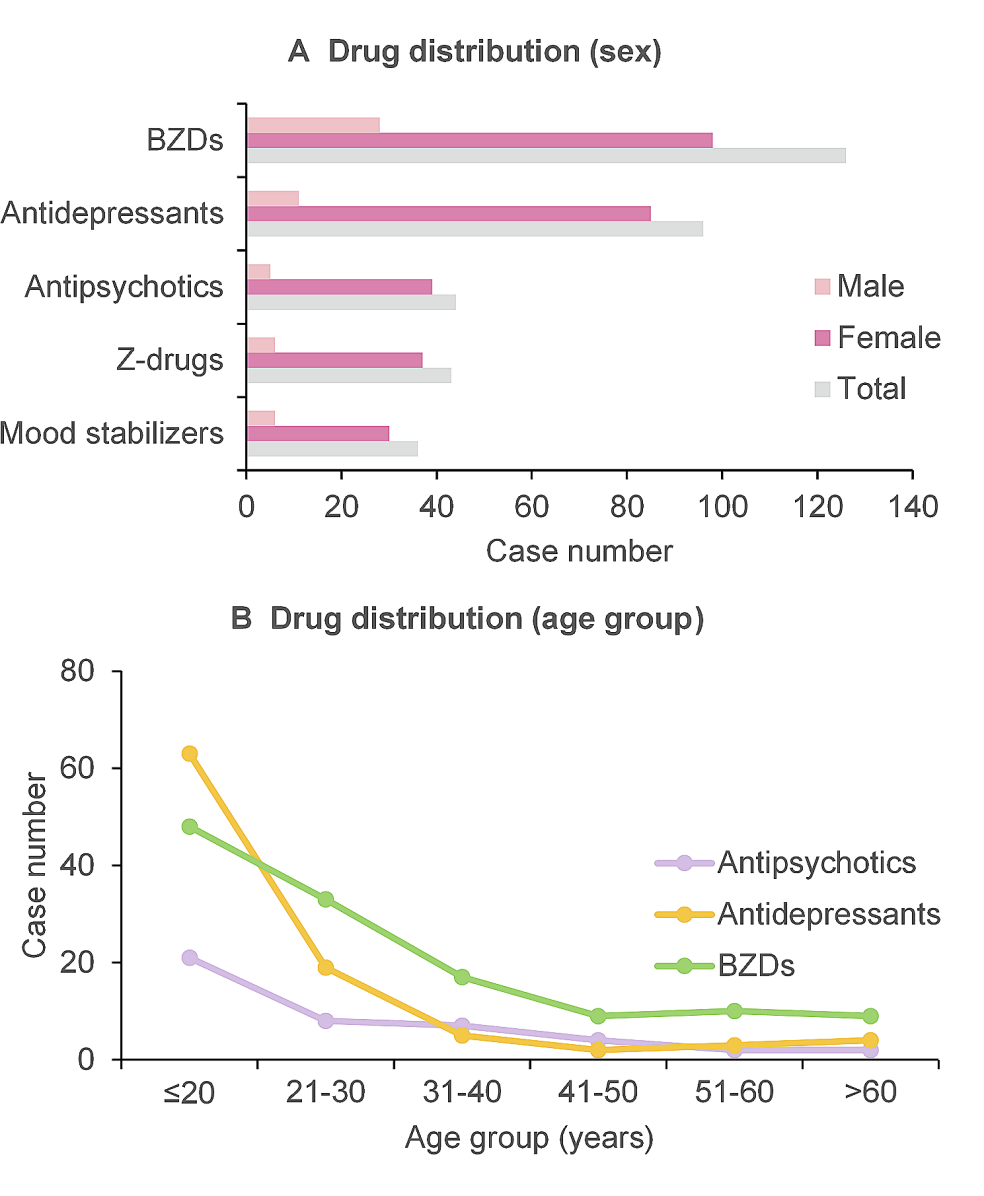
Drug distribution by (**a**) sex and (**b**) age group. (BZDs, benzodiazepines)

**Fig. 3:**
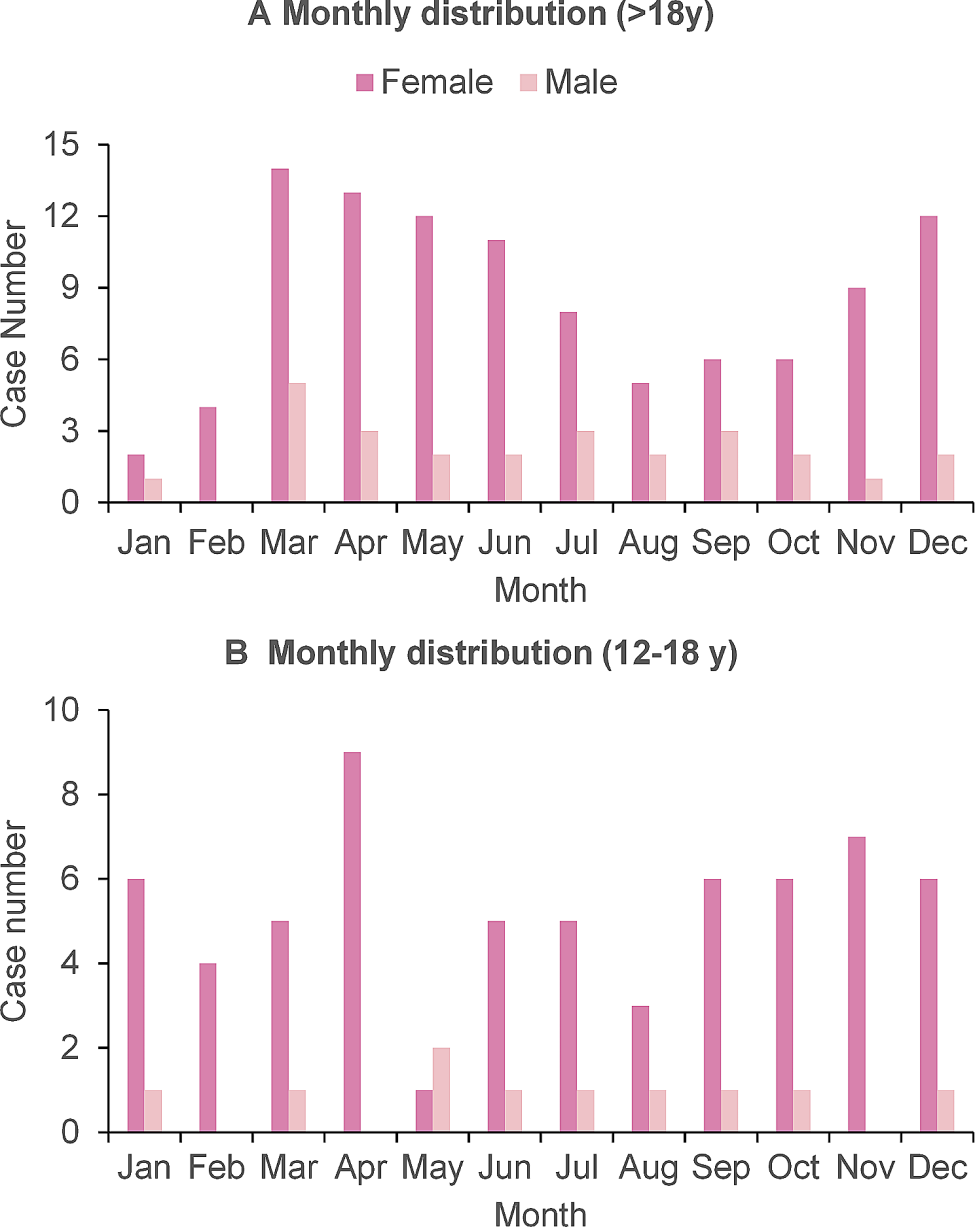
Monthly distribution by (**a**) total and (**b**) 12–18 years old

BZDs remained the most prevalent drug across all age groups except for adolescents, where antidepressants were the most common. Notably, BZDs and Z-drugs were significantly less common among adolescents compared to adults (Table 2; Fig. 2). The monthly distribution of ED presentations for adolescents revealed a peak in absolute caseload during April, followed by declines in May and August and another peak in November (Fig. 3). A similar trend was observed in the adult group.

**Table 2:** The main characteristics of patients who presented due to attempted suicide with drugs by age group

Variables	Number of Cases (%)		*P*
	Adolescents (*n* = 73)	Adults (*n* = 128)	
Female	63(86)	102(80)	0.240
Medication time^*^			0.605
Daytime	32(55)	42(49)	
Early-night	12(21)	24(28)	
Late-night	14(24)	20(23)	
Medication on weekends/holidays	14(19)	32(25)	0.345
Concomitant use of alcohol	0(0)	10(8)	**0.035**
≥ 3 drugs consumption	17(23)	25(20)	0.529
Aftercare: hospitalization	33(45)	59(46)	0.903
Psychotropic drugs			
BZDs**	38(52)	88(69)	**0.019**
Antidepressants	52(71)	43(34)	**< 0.001**
Antipsychotics	17(23)	27(21)	0.718
Z-drugs	6(8)	37(29)	**0.001**
Mood stabilizers	16(22)	20(16)	0.263
Nonpsychotropic drugs	18(25)	21(16)	0.155

*57 cases missing; **BZDs, benzodiazepines

Approximately 41% (*n* = 82) of the patients received a single-drug overdose, while the majority (55%; *n* = 110) received multiple drugs. Specifically, 68 (34%) patients took two drugs, and 42 (21%) took three or more drugs. Although, daytime on weekdays was the predominant time at which drugs were ingested, there was no significant differences among medication time. Hospitalization for further treatment occurred in approximately half of the patients who presented to the ED. Comparisons between hospitalized and nonhospitalized patients did not reveal significant differences in sex, age, or medication time (Supplementary Table 1). Significantly different patterns of substance use were identified between the adult and adolescent groups. Adolescents were more likely to overdose on antidepressants compared to adults (*P* < 0.001). Conversely, they were less likely to overdose on BZDs (*P* = 0.019) or Z-drugs (*P* = 0.001). Concomitant use of alcohol was not observed in the adolescent group.

## Discussion

This study investigated the characteristics and patterns of drug overdose suicide attempts among individuals seeking emergency care at a psychiatric ED during the COVID-19 pandemic. Our findings suggest that individuals presenting to the ED with drug overdose are more likely to be females, with BZDs being the most common overdose drug, followed by antidepressants and antipsychotics. Among younger females, antidepressants were the most common overdose drug.

### Common in females

Our results align with previous studies conducted in both general and psychiatric hospital EDs, which indicate that females constitute the majority of suicide attempts [4, 5, 9, 12, 13]. This highlights a relatively stable pattern of drug overdose suicide attempts. There might be two potential explanations exist for these patterns. (1) Gender differences in suicidality: Despite evidence suggesting a significant decline in overall suicide (mortality) rates in China over the past decades, particularly among females [14, 15], subgroup analyses revealed a concerning trend. No significant change was observed in the 15–24 year age group, and a substantial increase in suicide mortality was identified in the youngest age group (5–14 years) [15]. Similarly, research from Shanghai reported a reversal in the downward trend of suicide rates between 2002 and 2020, with a concurrent rise in depression diagnoses among suicide victims [16]. Additionally, several studies suggest a potentially heightened risk for suicidality among females during adolescence [17–21]. Our findings support this, with a fourfold greater rate of drug overdose suicidality in females than in males. (2) Impact of COVID-19: The pandemic may have contributed to this increased risk. While initial reports suggested relatively stable or declining suicide rates during the early months of the pandemic [22, 23], a growing body of evidence from systematic reviews and meta-analyses indicates an overall upward trend in suicidal ideation and attempts throughout the pandemic [24–30]. Notably, some research suggests a heightened vulnerability among young females, who may have experienced a more substantial increase in psychological distress compared to males [25, 27–29, 31–35]. However, some recent studies indicate that males might be more heavily impacted [36, 37].

### Risk factors in a psychiatric ED setting

This study also offers unique insights specific to a psychiatric ED setting. Notably, more than 80% of patients presented with overdoses of psychotropic drugs, suggesting that they had preexisting diagnoses of mental disorders and were already receiving medication. It is plausible that individuals attempting suicide without a diagnosed mental illness may be less inclined to seek help from a psychiatric ED. This aligns with the observation that even individuals with diagnosed mental illnesses may avoid seeking psychiatric care due to the stigma associated with mental disorders [38–40]. 

A retrospective study of psychiatric patients who attempted suicide and received emergency care at a general hospital revealed that major depressive disorder was the most prevalent diagnosis associated with the suicide attempts [9]. While definitive diagnoses cannot be established within the limitations of our ED setting, it is reasonable to hypothesize that depression may be the most prevalent underlying mental disorder among younger patients, particularly considering antidepressants were the most common overdose drugs in this age group. This observed association between depression and suicide attempts in our psychiatric ED aligns with findings from previous studies conducted in high-income Western settings [10, 41–43]. However, it is noteworthy that earlier research in China by Phillips et al. [44] reported a lower overall rate of mental disorders (63%) among completed suicides. Additionally, a meta-analysis focusing on low- and middle-income countries suggests that only 45% of individuals engaging in nonfatal suicidal behaviors have a diagnosed psychiatric disorder [45]. 

On the other hand, the prevalence of BZDs, commonly used for treating anxiety, as the most frequent overdose drug in our study suggests anxiety disorders may be a significant mental health concern within this ED setting. This aligns with network analyses [46] examining the relationships between depressive and anxiety symptoms and suicidality in adolescents during the COVID-19 pandemic, which found that both depression and anxiety are prevalent and contribute to suicidal ideation and attempts.

The increasing prevalence of mental health issues among younger people, particularly the high incidence of nonsuicidal self-harm in teenagers [47–49], aligns with our finding that younger individuals constitute the majority of those attempting suicide by drug overdose. This finding coincides with previous research indicating that the school year represents a vulnerable period for children and adolescents [13], highlighting the crucial role of school-based mental health support and intervention strategies. The observed seasonal pattern, with suicide peaks in April and November, may be explained by two potential mechanisms. First, it could align with the established pattern of increased stress during school hours [18]. Second, it might reflect a seasonal variation in the underlying mental health disorders themselves [50, 51]. Further research is necessary to elucidate the precise mechanisms underlying this seasonal pattern.

### COVID-19 pandemic effect

The emergence of COVID-19 has presented a complex and evolving challenge for mental health, but its impact on suicidal behaviour remains under investigation. While some studies, such as that by Irigoyen-Otiñano et al. [52], have suggested a temporary reduction in suicides during initial lockdown periods, a number of meta-analyses have documented increases in suicidal ideation and ED visits related to self-harm during the pandemic [24–29, 53–56]. Our findings align with the latter observations, demonstrating a rising trend in drug overdose-related ED visits during the pandemic period.

### Practical significance

Identifying the distinct characteristics of drug overdose suicide attempts among psychiatric patients holds direct relevance for improved emergency care practices. Clinicians should be particularly vigilant in assessing young female patients receiving prescriptions for antidepressants and/or BZDs, as our study highlights this group as particularly vulnerable. These findings underline the urgency of implementing targeted interventions in psychiatric ED settings, such as implementing suicide risk assessment protocols tailored to address the specific challenges of drug overdose suicide attempts, for example, educating family members on safe medication storage, and more importantly, enhancing suicide risk assessment prior to prescribing medication and implement evidence-based interventions for individuals with suicide ideation. These interventions may include safety planning, dialectical behavior therapy, or cognitive behavioral therapy, as feasible [57, 58]. Ketamine may also be a promising therapeutic option for suicidal ideation in both adolescents and adults, supported by emerging evidence from controlled trials [58–60]. 

### Strengths and limitations

While our study offers valuable insights from a psychiatric hospital ED, it is important to acknowledge limitations such as the relatively small sample size and potential for recall bias. Given that our study period encompasses only a portion of the COVID-19 pandemic (2020–2023), our findings may not fully capture the entirety of the pandemic’s impact. Therefore, caution is warranted when interpreting our results in the context of the broader pandemic. Furthermore, the lack of prepandemic data precludes definitive conclusions about changes in suicide patterns associated with COVID-19. Additionally, incomplete information on clinical features hinders a more comprehensive understanding of associated risk factors. Future research should aim to address these limitations by (1) gathering data on potential risk factors associated with suicide. (2) Utilizing larger, representative samples to enhance generalizability. (3) Employing longitudinal study designs to capture temporal trends in suicide patterns. Our ongoing longitudinal cohort study, which draws data from the Beijing Anding Hospital Mental Health Big Data Platform, aims to address these issues by spanning more than ten years and providing a more comprehensive picture of suicide patterns within the context of psychiatric ED settings.

## Conclusion

This study identified young females prescribed antidepressants and/or BZDs as a high-risk group for drug overdose suicide attempts in psychiatric EDs. These findings contribute to the evolving understanding of this complex phenomenon and emphasize the need for further research and improved clinical practices to ultimately improve patient outcomes and address this critical public health issue.

### Electronic supplementary material

Below is the link to the electronic supplementary material.

Supplementary Material 1

## Data Availability

The corresponding author will consider the data sharing requests on a case-by-case basis following relevant ethical and privacy regulations.
