# Supplementary material for: Characteristics of drug overdose suicide attempts presenting to the psychiatric emergency department of Beijing Anding Hospital
Source: BMC Public Health. 2024 Jun 14;24:1597. doi: 10.1186/s12889-024-19095-4 (PMC11179331; doi:10.1186/s12889-024-19095-4)
Supplement: Supplementary file 1 — Supplementary Material 1 [file 12889_2024_19095_MOESM1_ESM.docx]

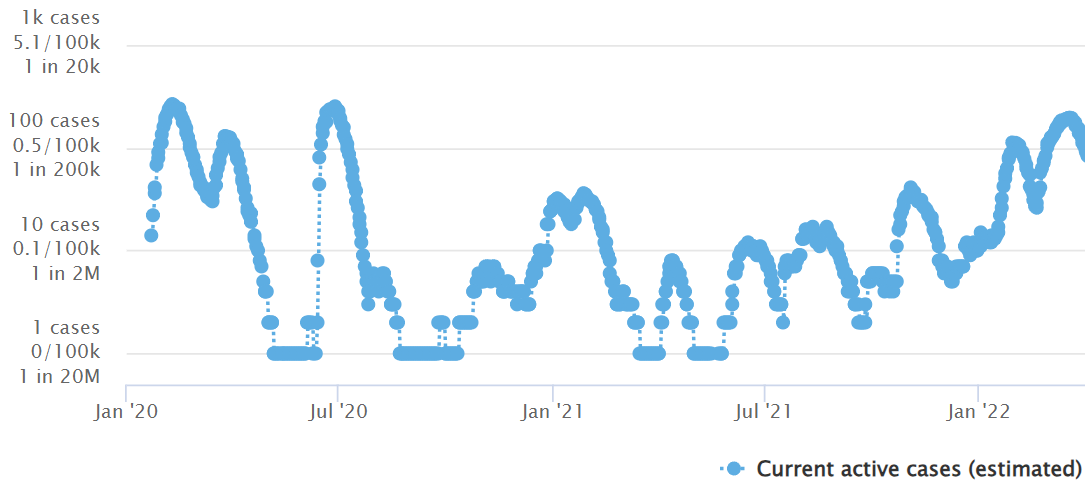


**Supplementary Figure** The graph shows the development of confirmed COVID-19 cases from January 2020 to January 2022 in Beijing. (Data Source: [Johns Hopkins University CSSE](Johns%20Hopkins%20University%20CSSE); <https://coronalevel.com/China/Beijing/> )

| **Supplementary Table** |  |  |  |
| --- | --- | --- | --- |
| The main characteristics patients who presented due to attempted suicide with drugs by aftercare. | | |  |
| Variables | Number of Cases (%) | | *P* |
|  | Hospitalization (n=92) | Outpatient (n=109) |  |
| Female | 74(80) | 91(84) | .574 |
| Age group (years) |  |  |  |
| Median (IQRs) | 23 (31) | 23 (17) | .303 |
| ≤20 | 42(46) | 48(44) |  |
| 21–30 | 16(17) | 33(30) |  |
| 31–40 | 7(8) | 13(12) | .066 |
| 41–50 | 9(10) | 4(4) |  |
| 51–60 | 10(11) | 6(6) |  |
| >60 | 8(9) | 5(5) |  |
| Medication time^*^ |  |  | .512 |
| Daytime | 33(49) | 41(54) |  |
| Early-night | 16(24) | 20(26) |  |
| Late-night | 19(28) | 15(20) |  |
| Medication on weekends/holidays | 16(17) | 30(28) | .088 |
| Concomitant use of alcohol | 5(5) | 5(5) | 1.000 |
| ≥3 drugs consumption | 18(20) | 24(22) | .670 |
| Psychotropic drugs |  |  |  |
| BZDs | 57(62) | 69(63) | .844 |
| Antidepressants | 44(48) | 52(48) | .883 |
| Antipsychotics | 19(21) | 25(23) | .696 |
| Z-drugs | 19(21) | 24(22) | .814 |
| Mood stabilizers | 13(14) | 23(21) | .199 |
| Nonpsychotropic drugs | 22(24) | 17(16) | .137 |
| *57 cases missing; IQRs, interquartile ranges ;BZDs, benzodiazepines. | | | |
